# Characterization of Nuclear Pleomorphism and Tubules in Histopathological Images of Breast Cancer †

**DOI:** 10.3390/s22155649

**Published:** 2022-07-28

**Authors:** Hayde Peregrina-Barreto, Valeria Y. Ramirez-Guatemala, Gabriela C. Lopez-Armas, Jose A. Cruz-Ramos

**Affiliations:** 1 Instituto Nacional de Astrofísica, Óptica y Electrónica, Luis Enrique Erro 1, Santa Maria Tonantzintla, San Andres Cholula 72840, Puebla, Mexico; hperegrina@inaoe.mx (H.P.-B.); yurasamai@inaoep.mx (V.Y.R.-G.); 2 Centro de Enseñanza Técnica Industrial, C. Nueva Escocia 1885, Guadalajara 44638, Jalisco, Mexico; glopez@ceti.mx; 3 Instituto Jalisciense de Cancerología, Coronel Calderón 715, Guadalajara 44280, Jalisco, Mexico; 4 Departamento de Clínicas Médicas, Centro Universitario de Ciencias de la Salud, Universidad de Guadalajara, Sierra Mojada 950, Guadalajara 44340, Jalisco, Mexico

**Keywords:** automatic classification, breast cancer diagnosis, digital image processing, histological differentiation grade

## Abstract

Breast cancer (BC) diagnosis is made by a pathologist who analyzes a portion of the breast tissue under the microscope and performs a histological evaluation. This evaluation aims to determine the grade of cellular differentiation and the aggressiveness of the tumor by the Nottingham Grade Classification System (NGS). Nowadays, digital pathology is an innovative tool for pathologists in diagnosis and acquiring new learning. However, a recurring problem in health services is the excessive workload in all medical services. For this reason, it is required to develop computational tools that assist histological evaluation. This work proposes a methodology for the quantitative analysis of BC tissue that follows NGS. The proposed methodology is based on digital image processing techniques through which the BC tissue can be characterized automatically. Moreover, the proposed nuclei characterization was helpful for grade differentiation in carcinoma images of the BC tissue reaching an 0.84 accuracy. In addition, a metric was proposed to assess the likelihood of a structure in the tissue corresponding to a tubule by considering spatial and geometrical characteristics between lumina and its surrounding nuclei, reaching an accuracy of 0.83. Tests were performed from different databases and under various magnification and staining contrast conditions, showing that the methodology is reliable for histological breast tissue analysis.

## 1. Introduction

Breast cancer (BC) is the abnormal and disorganized growth of cells in the breast tissue, and according to the World Health Organization (WHO), it is the most common type of cancer in women. Therefore, the survival prognosis of a woman diagnosed with breast carcinoma is directly related to tumor behavior. A pathologist analyzes a portion of tissue under a microscope to establish the histopathological grade of cellular differentiation [1]. This evaluation is made by describing the variations and abnormalities of the structures in the tissue [2]. For this purpose, the histological Nottingham Grading System (NGS) considers three morphological characteristics of the breast tissue to describe the grade of differentiation: tubular formation, nuclear pleomorphism, and mitotic count [3,4,5]. However, this task implies that the description of the characteristics depends on human perception, and, even under a trained eye, it is subject to human error in the diagnosis [6]. For this reason, developing support tools, such as Computer-Aided Diagnosis (CAD) systems, may help carry out diagnostics. Moreover, automatic analysis of the breast tissue image could help compare and support diagnosis results.

Usually, histological evaluation is performed through a quantitative feature analysis based on the NGS. Several strategies have been developed to detect and quantify cellular structures in histopathological images automatically. Naik et al. [7] used an automatic Bayesian to classify healthy and cancerous tissue based on the analysis of features, such as intensity values and relationship among nuclei pixels, reaching an accuracy of 80%. However, analysis of those features depended on manual segmentation of the centroid of nuclei, a time-consuming task. The study of healthy and cancerous tissue may generate a large set of features, although not all have the same relevance. Doyle et al. [8] used spectral clustering to identify common features in nuclei, which simplified the set of textural features used as input in the Support Vector Machine (SVM) classifier, reaching an accuracy of 93%.

Additionally, high-frequency features provide information associated with the differentiation grade in nuclei. For instance, high-frequency information has been extracted through wavelet transform [9] or active contours [10] that identified relevant differences in intensity. Comparison between pair of grades (e.g., G1–G2/G1–G3) reached accuracy levels up to 93%, but comparison among the three grades was significantly lower with a maximum accuracy of 74%. Automatic feature extraction also has been applied by employing Deep Learning (DL) [11,12], although for the three differentiation grades the reached accuracy was 69%. However, extracted features could not be related to the reference NGS. Given the complexity of identifying characteristics from the NGS, fewer reports analyzed more than one characteristic in the histological tissue. Petushi et al. [13] studied nuclear pleomorphism and tubule formation by integrating automatic feature extraction algorithms and classifiers. An accuracy of 72% was achieved by considering three grades of differentiation. To the best of our knowledge, only Dalle et al. [14] have addressed the three differentiation grades, including all the characteristics stated in the NGS. The authors proposed a multi-resolution approach that improves the analysis of nuclear pleomorphism and mitotic count in high-resolution images while analyzing tubule formation in low-resolution images. Nevertheless, an evaluation of the features used in the tissue classification process was not reported.

In the analysis of related works, two main issues are noticed. First, histopathological images usually vary in intensity or stain conditions; texture or high-frequency features may change among sets acquired under different conditions. Secondly, feature extraction should not depend on the intrinsic characteristics of the image but on the elements that the experts have identified as relevant. Therefore, the classification accuracy and the relation of analyzed features with those used in the practical histological analysis are important in a diagnostic support system.

This work proposes a quantitative analysis of the relevant structures in histological sections of BC with Hematoxylin and Eosin (H&E) staining to characterize the grade of cellular differentiation based on two of the characteristics considered in the NGS: nuclear pleomorphism and tubular differentiation. An initial advance reported in [15] addressed clumps of cell nuclei segmentation since these may affect the counting and analysis when considered as one single element. The extended methodology proposed here shows that it is possible to differentiate between healthy and cancer nuclei based on the analysis of shape characteristics. Moreover, these characteristics distinguished among the three differentiation grades in carcinoma images. Additionally, an analysis of the relation between a lumina region and the surrounding nuclei that more likely define a tubule was proposed. The obtained results for grade differentiation reached an accuracy of 0.84 based on nuclear pleomorphism and 0.83 based on tubules identification.

This document describes the basic concepts of the proposed methodology in Section 2. The proposed methodology for analyzing, segmenting, and characterizing tissue structures is explained in Section 3. Section 4 describes the experiments carried out and the obtained results. Finally, conclusions about the findings are presented in Section 5.

## 2. Materials and Methods

### 2.1. Differentiation Grade in Histopathology

**Figure 1:**
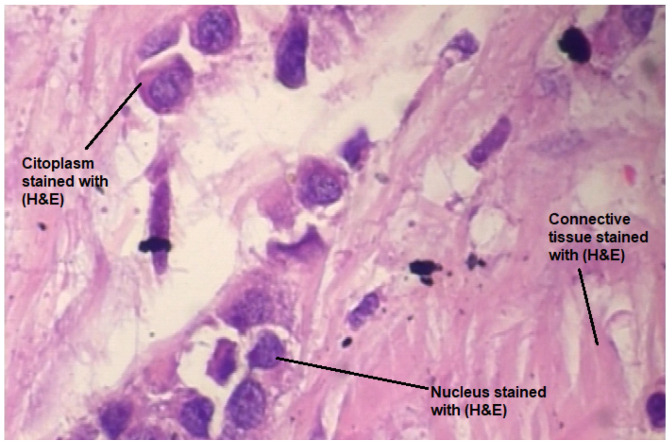
Sample of a digital histological image of lobular carcinoma stained with H&E from the BreakHis database [18].

According to the Royal College of Pathologists, histopathology studies structures in tissues characteristic of disease in order to reach a diagnosis. A tissue sample (biopsy) is obtained from the body tissue and examined under microscopy after being prepared and fixed on glass slides. Often, the cellular structure of tissue components is intended to be highlighted with different colorants. The H&E staining remarks nuclei in purplish-blue while cytoplasm and extracellular matrix in pinkish tones. (Figure 1) [16]. Given the usefulness of the (H&E) staining, it has become a standard for tissue analysis [17].

The grade of cellular differentiation describes the morphological variations of the mammary tissue, i.e., how a tissue varies from the appearance of normal tissue. Thus, the differentiation grade is associated with the malignancy degree in the tissue, and based on it, the pathological diagnosis guides the most appropriate treatment. The NGS [2], the standard in histological grading, considers three cellular characteristics in the tissue to determine the grade of differentiation: tubular formation (structure corresponding to the milk ducts), nuclear pleomorphism (deformation of nuclei), and mitotic count (nuclei that are in the process of mitosis).

### 2.2. Mathematical Morphology

Mathematical morphology is a set of image processing techniques useful in describing image structures. A morphological transformation requires structures (objects) in the image *I* to be processed by a structuring element *B*, which imposes the configuration or morphology (shape) used for such processing [19]. Although a square shape is typically used, the shape of the structuring element may vary depending on the objects of interest (circular, diamond, line) [20]. Opening and closing (Equations (1) and (2)) are basic morphological operations based on morphological erosion $\varepsilon_{\lambda B}\left(I\right)\left(x\right)=min\left\{I(y);y\in \lambda B\right\}$ and dilation $\delta_{\lambda B}\left(I\right)\left(x\right)=max\left\{I(y);y\in \lambda B\right\}$, where λ indicates the scale or size of *B*. A morphological dilation enlarges the area of image objects while morphological erosion contracts them. However, avoiding a significant change in object size could be important for some processes. Then, the dual transformation is applied as a second step to avoid such effects, e.g., dilation after erosion. In this sense, the purpose of the second transformation in a morphological opening or closing is to restore the object size affected by the first transformation to some extent.

(1) $$\gamma_{\lambda B}\left(I\right)=\delta_{\lambda B}\left[\varepsilon_{\lambda B}\left(I\right)\right]$$

(2) $$\varphi_{\lambda B}\left(I\right)=\varepsilon_{\lambda B}\left[\delta_{\lambda B}\left(I\right)\right]$$

### 2.3. Granulometry

Granulometry is an operation, based on morphological openings, that allows measuring the effect that a scale λ has over the grains in the image; thus, λ can be related to the size of the grains. In digital image processing, grain refers to a structure or object associated with a certain gray level (solid) [21]. Thus, grain size analysis is a tool for estimating the sizes of relevant objects in an image. Granulometry consist of a set of operations $\left\{\gamma_{\lambda }\right\}$. Then, the granulometry of image *I* consists of mapping *I* through morphological apertures in $\left\{\gamma_{\lambda }\right\}$, and measuring the effect of λ+Δ in the image through a normalized difference between $mes(\gamma_{\lambda }\left(I\right)$) and $mes(\gamma_{\lambda +\Delta }\left(I\right))$, where mes is the accumulated sum of gray levels. The measured effect is called pattern spectrum (PS(I)) (Equation (3)) [22]. Then, it is assumed that a size λ+Δ generates a high impact when it matches the size of representative structures in the image.

(3) $$PS\left(I\right)=\frac{mes\left(\gamma_{\lambda }\left(I\right)\right)-mes\left(\gamma_{\lambda +\Delta }\left(I\right)\right)}{mes(I)}$$

### 2.4. Watershed

**Figure 2:**
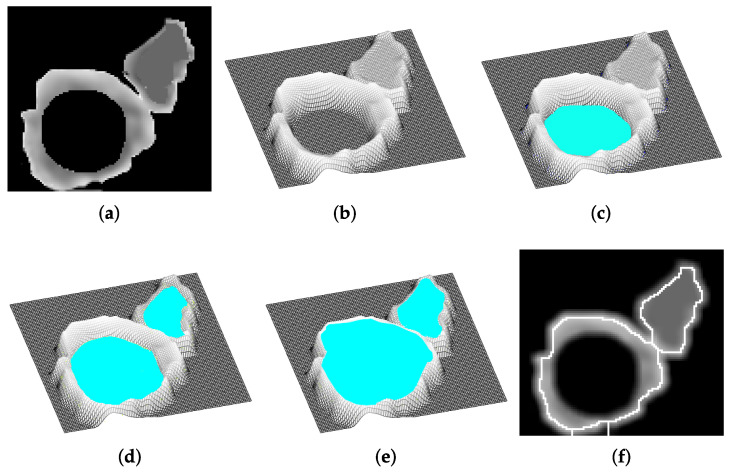
Watershed flooding process for region segmentation. (**a**,**b**) Original image and its representation as a topological map, (**c**,**d**) flooding starting from local minima to higher levels forming catchment basins, (**d**,**e**) local maxima reached stopping flooding, and (**f**) the final borders indicating the division of regions.

Watershed is a segmentation technique that enables the dividing of regions in the image by considering the increment of intensity values from a local minimum to the maximum local change achieved. Watershed labeled pixels by considering their spatial proximity, gray level gradient, or texture [19,23]. Watershed analyzes the image as a topological map where a local minimum (lower intensity) corresponds to the bottom of a catchment basin (Figure 2a,b) from where the flood starts. The flood refers to marking the analyzed pixels under the same label to indicate that they belong to the same region (Figure 2c,d). When a local maximum (higher intensity) is reached, the flood stops to avoid overflowing into another region, i.e., duplicate labeling (Figure 2e). Thus, limits among basins can be drawn, generating the segmentation of objects (Figure 2f). Then, the watershed of image *I* in the space *D*, containing a set of local minimums ${\left\{m_{k}\right\}}_{k\in I}$, is defined by the intersection between a set of points $CB\left(m_{i}\right)=\left\{x\in I|x\;\mathrm{is}\;\mathrm{closer}\;\mathrm{to}\;m_{i}\;\mathrm{than}\;\mathrm{to}\;\mathrm{any}\;\mathrm{other}\;m_{j}\right\}$ and the set of points in *D* (Equation (4)) [24].

(4) $$Wshed\left(I\right)=D\cap {\left(\underset{i\in I}{⋃}CB\left(m_{i}\right)\right)}^{c}$$

### 2.5. Circularity Estimation

Circularity metrics allow measuring how circular a shape is. In some kinds of images, it is relevant to identify regions of interest with higher circularity since they could be associated with specific particles or characteristics of the sample [25]. The circularity measure is often used in diverse applications, such as medicine and industrial processes [26,27,28]. Although regions of interest could be perceived as circular, in real samples, they often present some degree of deformation, fold, or defect caused by the nature of the process (e.g., occluded or incomplete regions) that may affect their quantitative assessment through a circularity metric. The MOR circularity metric [29] is based on the probability distribution of radius f(r) from the center of the region *c* to its edge pixels *r* and assumes that the set *r* does not follow a Gaussian distribution as the object is deformed, making MOR less sensitive to distortions in the analyzed region. Thus, MOR is defined as the ratio between the area centered in the $r_{i}$ of higher probability in f(r) and the total area (Equation (5)), where $k_{1}$ and $k_{2}$ are the local minimums of $r_{i}$.

(5) $$MOR=\frac{\int_{k_{1}}^{k_{2}}f\left(r\right)dr}{\int_{-\infty }^{+\infty }f\left(r\right)dr}$$

## 3. Methodology

The proposed methodology comprises three main parts: (i) identification and segmentation of tissue structures corresponding to nuclei and tubules, (ii) feature extraction and analysis, and (iii) classification of nuclear pleomorphism and tubules quantification. This methodology is based on digital image processing techniques, which allow analyzing the morphology of the elements of interest in the histological tissue.

### 3.1. Nuclei Extraction

The first part of the methodology comprises an analysis of the elements or structures contained in the image to identify those corresponding to nuclei. For this, an analysis of structure sizes and filtering by area is used to determine the most likely structures of interest. Additionally, an image contrast enhancement is applied to distinguish better among elements in the image. Histopathological images usually have an H&E stain highlighting the tissue components in purple tones, darker in the nucleus area, and pink in the cytoplasm and related tissue surrounding the nucleus. Given that the main contribution in these tones comes from the red color, the R channel will be used in the following processes. A first approximation of this step was addressed in [15] and is now extended as part of the proposed methodology.

#### 3.1.1. Separation of Tissue and Nuclei

For this work, a histopathological image is divided into elements of interest (nuclei and tubules) and background (connective tissue and empty parts of the slide). However, H&E stain conditions may vary from one image to another, affecting contrast among the elements in the image. Then, a contrast enhancement is required to facilitate later nuclei extraction. Gamma correction is often used in contrast enhancement since it is related to the distribution of values in which human vision perceives stimuli better. A contrast adjustment by the Gamma correction is defined as the power function $V_{out}=AV_{in}^{g}$, where $V_{in}$ is a real positive value, *A* is a constant, and *g* is the gamma encoding that symbolizes a numeric parameter [30]. This correction improves contrast by mapping the current range of values in the image $\left[low_{in},\;high_{in}\right]$ to a wider range; for instance, in an 8-bit image the complete range of values is [0,255]. The performed contrast enhancement was based on a linear correction (g=1) considering that $low_{in}=\mu -\left(\sigma *2\right)$ and $high_{in}=\mu$, where μ and σ are the mean and standard deviation of values in the image. In this way, it is expected that objects of interest be highlighted.

Nevertheless, it must be noted that contrast conditions may vary in regions of the same image. Therefore, if a general adjustment takes statistics from the whole image, some objects of interest may be lost. For example, cell nuclei on low contrast areas could take a value similar to the background, making their subsequent segmentation difficult.

**Figure 3:**
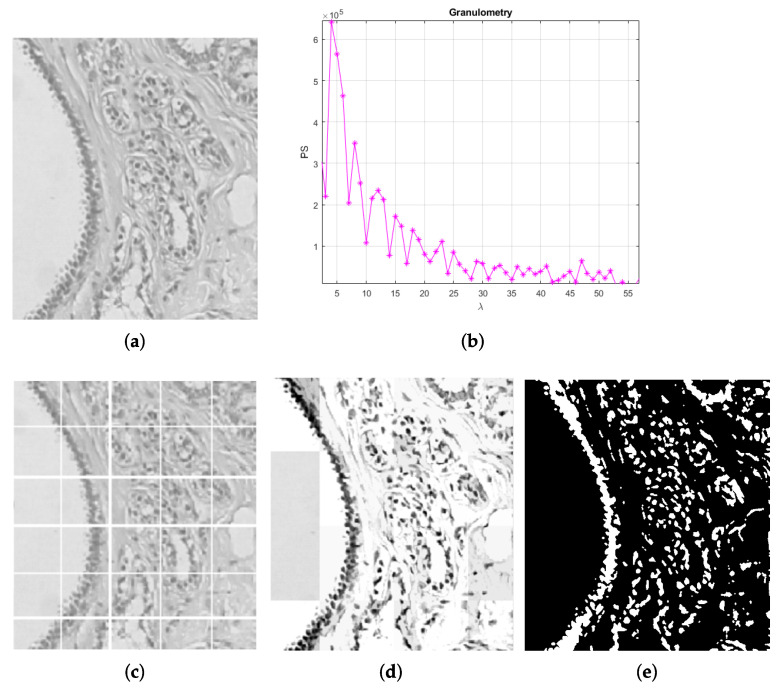
Process to separate tissue and nuclei [15]: (**a**) original image, (**b**) granulometric analysis, (**c**) separation by sub-regions using $\lambda_{n}$ with higher PS as a reference, (**d**) local contrast adjustment, and (**e**) sub-regions binarization.

For a contrast enhancement suitable to the particular stain conditions of histopathological images, the image is divided into sub-regions where values adjustment depends on local conditions. It must also be considered that histopathological images may be acquired at different magnification and, depending on it, elements in the image are observed to be smaller or larger. Therefore, a sub-region size must ensure to contain the elements of interest. As a reference, the image’s most representative size is considered associated with the elements of higher occurrence, i.e., nuclei. A granulometric analysis with a circular structural element is performed since cells tend to be rounded shapes (Figure 3a,b). As observed, most elements are reached by $\lambda_{n}=4$, meaning that nuclei cover an area of 7×7. After heuristic testing, it was determined that square sub-regions of size $25\lambda_{n}$ were suitable for contrast analysis (Figure 3c). In this way, the sub-division of the image is independent of magnification.

Once the image has been divided, local contrast adjustment is performed in each sub-region by considering their statistics. As observed in Figure 3d, nuclei are highlighted according to their local surrounding; where nuclei are similar to the rest of the tissue, Gamma correction generates a higher distance of gray values, increasing the contrast. This process facilitates nuclei binarization with Otsu’s method, eliminating the background and other tissue parts that are not of interest. Figure 3e shows how nuclei have been separated from the rest of the information in the image.

#### 3.1.2. Nuclei Segmentation

**Figure 4:**
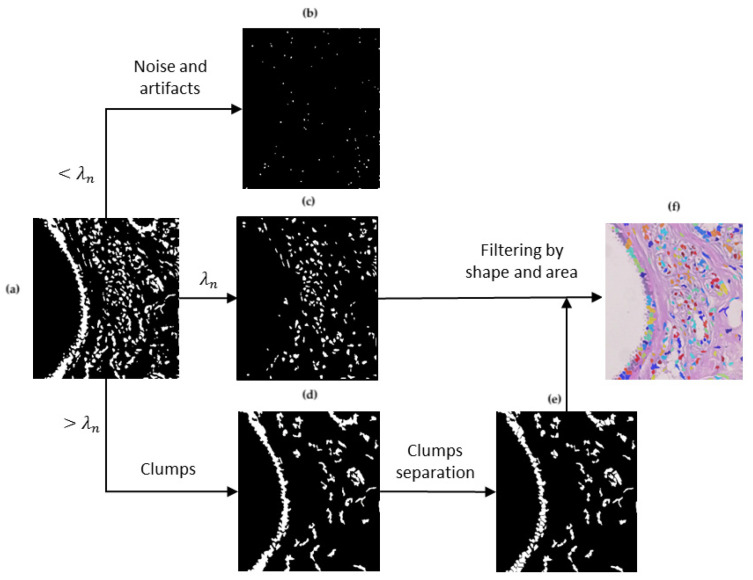
Cell nuclei segmentation and filtering. (**a**) Binary cell nuclei markers, (**b**) small elements (<$\lambda_{n}$), (**c**) elements of average size ($\lambda_{n}=4$), (**d**) clumps of cells (>$\lambda_{n}$), (**e**) clumps division, and (**f**) final segmentation fusion.

The previous binary image provides a partial segmentation of nuclei. However, nuclei cells have not been segmented yet. According to the granulometric analysis, elements of interest have a size $\lambda_{n}=4$ and must be preserved. On the one hand, it is assumed that elements of a smaller size (<$\lambda_{n}$), associated with noise or artifacts, are not relevant and must be removed from the image. On the other hand, elements of a larger size (>$\lambda_{n}$) are considered clumped cells of possible interest and should be separated for later individual analysis. The pre-selection of elements is shown in Figure 4a–d.

Clumps of cells may contain a significant number of nuclei that, if eliminated, could affect the histopathological image assessment. Then, it is relevant to identify the inner nuclei that these components contain and divide them. For this, a watershed transformation is applied, allowing individual identification of elements (Figure 4e). Then, all the elements in Figure 4c,e are put together in a single image. It must be remarked that not all segmented elements correspond to nuclei since some remaining clumps could not be divided by watershed. Segmentation results were improved through two filtering criteria based on the features that elements in the same image contain: nuclei tend to have similar areas and be rounded. By taking as reference the size $\lambda_{n}=\arg\max\left(PS\right)$ from granulometric analysis, a range of sizes is settled as $T_{\lambda }=[\lambda_{n}/2,\;\arg\max_{\lambda_{n}+1\leq x\leq X}\left(PS\left(x\right)\right)$, where *X* is the maximum value of λ analyzed in PS. This means that elements of interest go from half size of $\lambda_{n}$ to the second relevant size in PS. Thus, potential artifacts, over-segmentation errors, or undivided clumps are discarded. The mean and standard deviation of roundness values, estimated with MOR, was computed to establish the circularity threshold $T_{c}=\mu_{c}-\sigma_{c}$. Only elements with roundness greater or equal to $T_{c}$ are kept. The final segmented nuclei are illustrated in color over the original histopathological image in Figure 4f.

### 3.2. Tubule Detection and Segmentation

**Figure 5:**
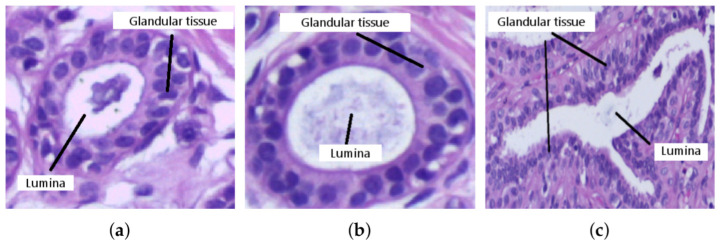
Tubules in breast tissue. Regular shaped tubule in (**a**) healthy tissue and (**b**) benign tumor tissue; and (**c**) irregular shaped tubule in cancerous tumor tissue. Images taken from [31].

The second part of the methodology is mainly focused on detecting tubules. A tubule has two main characteristics: the presence of an element of high intensity (lumina) surrounded by an area of connective tissue and nuclei (glandular tissue) that usually retain regularity in its distribution and orientation (Figure 5). In addition, the tubule commonly has high circularity, although this condition varies in cancerous tissues. Therefore, for the detection and segmentation of tubules, the proposed methodology consists of three steps: detecting lumina candidates, retrieving glandular tissue, and analyzing glandular tissue.

#### 3.2.1. Detection of Lumina Candidates

**Figure 6:**
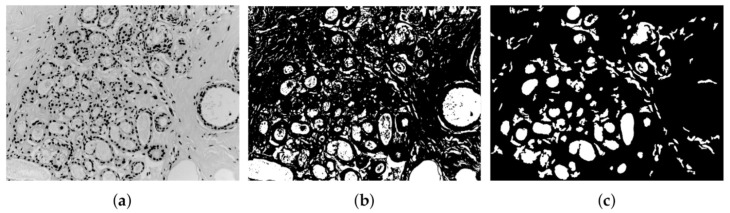
(**a**) Remaining connect tissue of benign tumor after nuclei segmentation [31], (**b**) lumina candidate segmentation, and (**c**) the refinement of luminas regions.

For the initial extraction of lumina candidates, the remaining tissue of previous nuclei segmentation is taken as a basis (Figure 6a) to avoid processing regions where a lumina is unlikely to be found. However, the remaining tissue may contain components resembling lumina and must be analyzed. A lumina tends to be a homogeneous region with a high intensity. Therefore, the mean intensity of connective tissue is taken as a threshold to isolate lighter regions (lumina candidates) from the rest of the connective tissue (Figure 6b). Once the initial extraction has been obtained, detected lumina candidates are analyzed and evaluated using two criteria. The first is an area criterion $U_{a}=2\left(Area_{n}\right)$, where $Area_{n}$ is the average area of nuclei in the image. It is assumed that lumina should be at least twice as long as nuclei. The second criterion is based on statistical mode and mean of intensity values. In the resulting lumina segmentation, there could be some elements that, despite having a high-intensity value, are not luminas. It is considered that a lumina candidate with mode below mean intensity could correspond to connective tissue but does not correspond to lumina; then, it must be deleted. Moreover, lumina candidates in the image edge are also deleted to avoid analyzing incomplete elements. The result of applying these two criteria is shown in Figure 6c.

#### 3.2.2. Retrieving of Glandular Tissue

**Figure 7:**
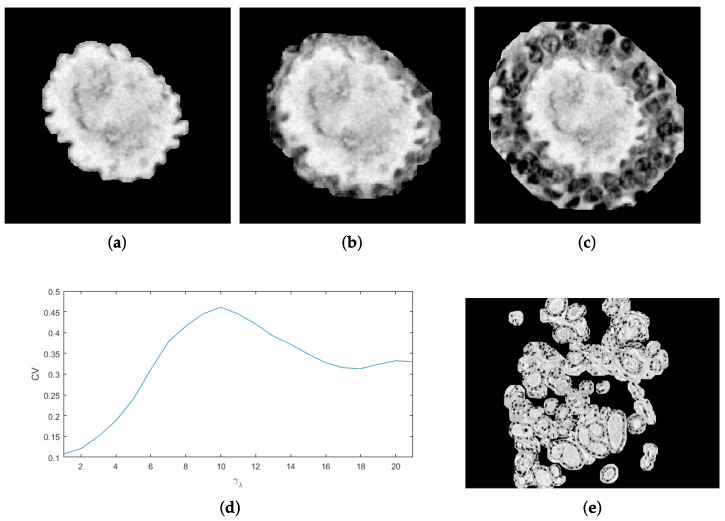
Retrieving glandular tissue through the CV measure and morphological dilations. (**a**) Original lumina candidate (CV=0.108), its increasing area after (**b**) $\gamma_{5}$ (CV=0.240) and (**c**) $\gamma_{10}$ (CV=0.460), and (**d**) the behavior of CV associated with the addition of nuclei; and (**e**) the retrieving of glandular tissue around lumina candidates after filtering with *d*.

Since the previous step focused on extracting the highest intensity regions, lumina candidates lack surrounding glandular tissue. Nevertheless, glandular tissue is a piece of essential information to confirm if the candidate corresponds with a tubule. The binary markers of luminas (Figure 6c) were thickened through a series of morphological dilations ($\gamma_{\lambda }$) to retrieve the glandular tissue area of interest. It is expected that the initial region (lumina candidate) grows to reach the glandular tissue containing the nuclei, defining a tubule (Figure 7). With each dilation, the inclusion of nuclei generates intensity changes that are considered to know when the region should stop growing. The variation coefficient $CV=\sigma_{I}/\mu_{I}$ is computed to assess the intensity change, where $\sigma_{I}$ is the standard deviation, and $\mu_{I}$ is the mean statistics; this metric helps to estimate the contrast in a region. For instance, Figure 7a shows a lumina candidate (initial region) that presents high values in most of its area; therefore, it is expected to have a low variation (CV=0.108). Figure 7b shows the growth of the region after being dilated five times, including part of the nuclei and increasing the variation of values, as CV=0.240 indicates. In Figure 7c, most of the nuclei surrounding lumina have been reached (CV=0.460). As the region is dilated and nuclei are reached, the intensity variation is expected to increase since nuclei are outlier values compared to the lumina values. This behavior is reflected in the plot of Figure 7d, where the CV value helps to evaluate if the region growth should continue. A decrease in CV indicates that the added area is mainly glandular tissue (high luminance values) or does not contain a relevant nuclei area anymore. Therefore, the increment in CV is used as a stop criterion for dilation.

It must be considered that some lumina candidates may not be related to a tubule since nuclei do not surround them. This condition is evaluated by considering the normalized mean values of the lumina ($\mu_{l}$) and the connective tissue added with dilation ($\mu_{\gamma }$). If the connective tissue includes a few nuclei or none, the difference $d=\mu_{\gamma }-\mu_{l}$ is small. On the other hand, it is expected that the inclusion of a significant number of nuclei generates a difference higher than *d*. Hence, if CV>d, it is assumed that the change in intensity is due to the presence of nuclei around the lumina candidate; otherwise, the lumina candidate is eliminated. As a result, lumina candidates with their corresponding glandular tissue are obtained. Figure 7e shows an example of the complete identification process of lumina candidates in a histopathological image of invasive carcinoma. Nevertheless, this result partially selects lumina candidates as possible tubules. Therefore, in the next step, glandular tissue is analyzed to identify tubules finally.

#### 3.2.3. Analysis of Glandular Tissue

**Figure 8:**
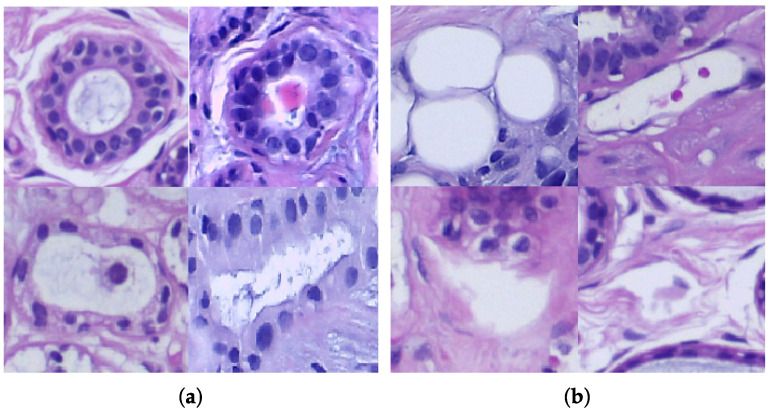
Light regions in the tissue: (**a**) related to lumina tubules and (**b**) other structures of similar composition that are not tubules. Images taken from [31].

Figure 8 shows light regions corresponding to luminas of tubules; some others correspond to gaps in the tissue or fatty regions. Therefore, the analysis of lumina candidates is relevant for identifying their possible association with tubules. This step addresses the surrounding nuclei distribution analysis to distinguish the tubules from the rest of similar structures.

Based on the previous segmentation of nuclei and lumina candidates (Section 3.1.2 and Section 3.2.2), it is possible to obtain their binary markers in the retrieved glandular tissue to be analyzed. For this purpose, some characteristics from the markers are analyzed to obtain more information about lumina candidates. First, it must be considered that the glandular tissue contains nuclei surrounding the lumina and some others from near unrelated regions. Since the count of nuclei can be relevant to identifying a tubule, it was established a minimum threshold considering that at least half of the lumina perimeter $p_{l}$ must be covered by the average diameter of the nuclei $d_{n}$ and the minimum distance among them $d_{min}$, i.e., $T_{n}=p_{l}/2\left(d_{n}+d_{min}\right)$. Therefore, candidates whose nuclei account is less than $T_{n}$ are unlikely to be a tubule.

**Figure 9:**
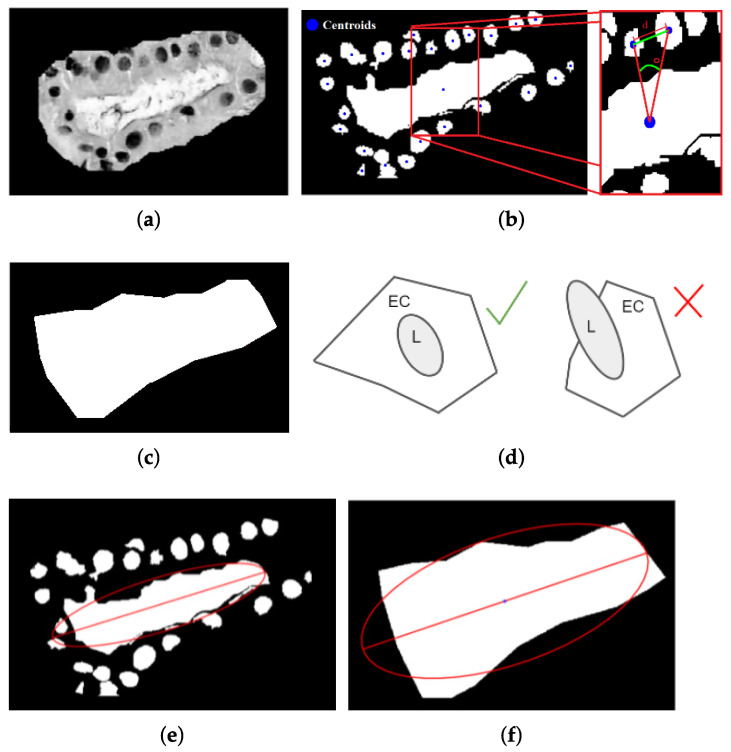
(**a**) Lumina candidate, (**b**) its binary markers of nuclei and lumina indicating their centroids, and (**c**) the envelope formed by nuclei. (**d**) The relationship of regions *L* and EC for tubule identification, and (**e**,**f**) the similarity between their respective ellipses.

**Figure 10:**
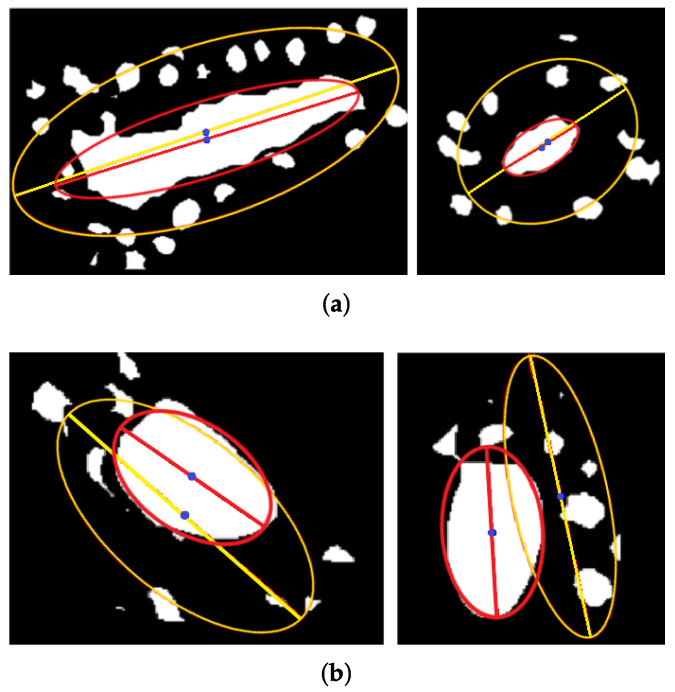
Lumina candidates description through the ellipses from their corresponding convex hull and centroid to distinguish between candidates (**a**) related to tubules and (**b**) related to other structures (no-tubules).

Secondly, although the number and distribution of nuclei may be different, it was observed that nuclei tend to be uniformly distributed around lumina (*L*), forming an envelope that is useful for characterizing tubules (Figure 9a). Three parameters are estimated to obtain the envelope: nuclei centroid, the distance between adjacent nuclei, and the angle between those and the lumina centroid (Figure 9b). From the nuclei and lumina centroids, the convex hull EC is estimated to represent them as an irregular polygon (Figure 9c). It is expected that the lumina associated with a tubule were completely contained in the convex hull formed by its nuclei (Figure 9d). Moreover, since most lumina candidates have an elliptical shape, it is assumed that EC and the lumina candidate regions can be described by elliptical properties, such as centroid and orientation. Then, also it is expected that ellipses from EC and lumina candidates are similar (Figure 9e,f). In Figure 10a, it is observed that the convex hull descriptions match with lumina candidates segmentation through their ellipses, having a slight difference when the lumina correspond with a tubule. On the other hand, when the lumina candidate is more likely a light region not related to a tubule, the ellipses present a higher difference Figure 10b.

**Figure 11:**
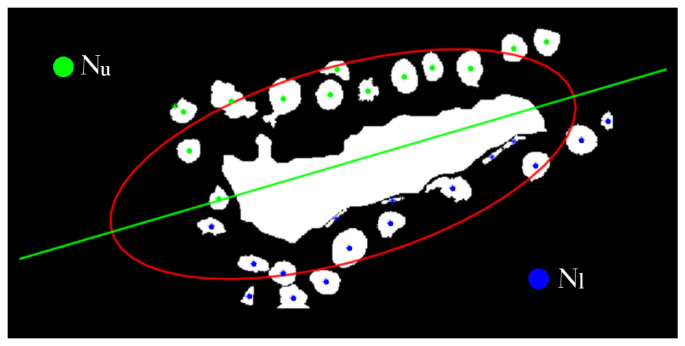
Separation of nuclei corresponding to $N_{u}$ and $N_{l}$ to perform the symmetry calculation.

Finally, symmetry in the distribution of nuclei around lumina is also a characteristic considered. For symmetry estimation, the major axis of the ellipse from EC is used as a reference. The general description of the tubule is associated with the ellipse from its convex hull since it includes both lumina and the nuclei around it. Then, the symmetry measure is obtained as $S=\min\left(N_{u},N_{l}\right)/\max\left(N_{u},N_{l}\right)$ where $N_{u}$ and $N_{l}$ are the counting of nuclei in the upper and lower side of the major axis of the ellipse, respectively (Figure 11). The measure *S* has values in the range [0,1], tending to 1 when there is a high symmetry. Thus, these three parameters ($T_{n}$, EC, and *S*) help find differences among lumina candidates that are more likely associated with tubules.

## 4. Results and Discussion

This section presents the results of histopathological images analyzed under the proposed methodology. Two main experiments were carried out: the differentiation degree based on nuclear pleomorphism and the identification of tubules. For this, several datasets were used and tested, as described below.

### 4.1. Dataset

Histopathological samples from three different databases were used to evaluate the proposed methodology. The Breast Cancer Image Classification (BreakHis) database [18] consists of images of 700×460 of breast benign (2480) and malign (5429) tumor tissue under different magnification factors (40×, 100×, 200×, and 400×). The Breast Cancer Histology database (BACH) [31] is composed of 400 microscopic images with a magnification factor of 200× and a resolution of 2048×1536. Samples in the BACH are divided into sets of 100 images in which tissue is labeled as healthy, benign tumor, carcinoma in-situ, and invasive carcinoma. The Breast Cancer Cell Segmentation (BCCS) database [32] contains 58 images of BC with a resolution of 896×768 pixels and is the only one that includes a ground truth of nuclei segmentation. These databases were used in the different stages of the proposed methodology to evaluate its performance, as described below.

### 4.2. Automatic Segmentation of Nuclei

For assessing the performance of the proposed nuclei extraction process, images from the BCCS were used since their ground truth allows a direct comparison of results. Segmentations obtained from the proposed nuclei extraction and the ground truth are denoted $S_{s}$ and $S_{t}$, respectively. For comparison results, a set of area-based segmentation metrics were used. Precision and sensitivity metrics were calculated as $\frac{TP}{TP+FP}$ and $\frac{TP}{TP+FN}$, respectively; where $TP=S_{s}\cap S_{t}$ are true positives, $FP=S_{s}-S_{t}$ are false positives, $TN=S_{t}-S_{s}$ are true negatives, $FN=E-S_{s}-S_{t}$ are false negatives, and *E* denotes the region, including all possible segmented regions. In addition, the Sørensen–Dice similarity coefficient was also calculated as $SDC=\frac{2\cdot TP}{2\cdot TP+FP+FN}$. The SDC value is in the interval [0,1], where 0 indicates that both segmentation are completely different and 1 suggests they are the same [33,34].

**Figure 12:**
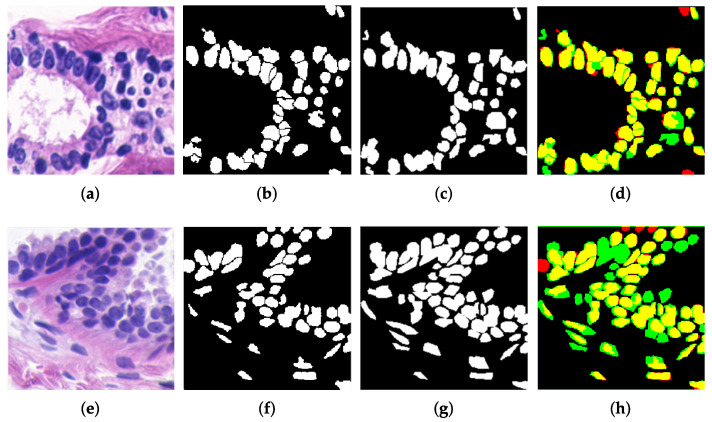
Evaluation of the proposed nuclei segmentation. (**a**,**e**) Original image, (**b**,**f**) proposed segmentation ($S_{s}$), (**c**,**g**) ground truth segmentation ($S_{t}$), and (**d**,**h**) segmentation comparison; $S_{s}$ in red, $S_{t}$ in green, and their coincidence in yellow.

**Figure 13:**
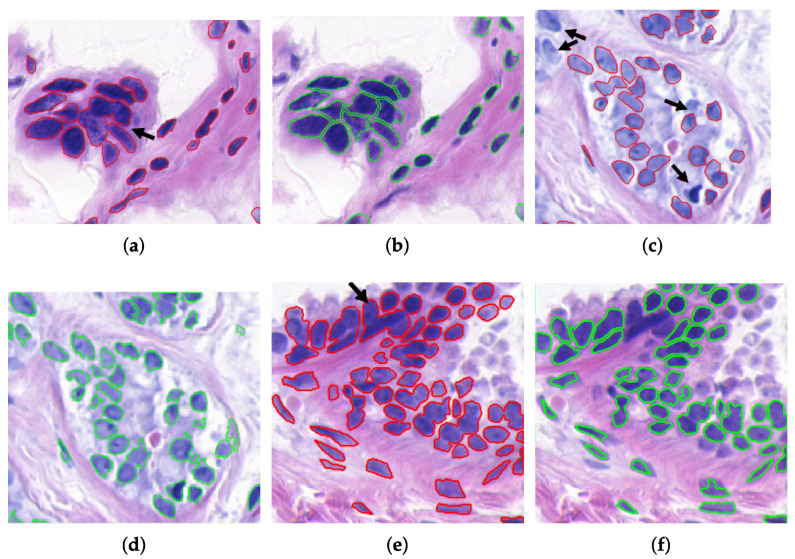
Differences were found between the proposed nuclei segmentation and the ground truth. (**a**,**b**) Separation of nuclei clusters, (**c**,**d**) nuclei detection, and (**e**,**f**) elimination of nuclei significantly different from the average features (shape/area) considered in the filtering.

Figure 12 shows some examples of original histopathological images and the comparison between their obtained segmentation (red), their corresponding ground truth (green), and the coincidence of both (yellow). Although a significant similarity can be observed between both results, also some differences affect metric values. It was identified that differences occurred due to three main factors: (i) the segmentation of the ground truth does not consider the separation of nuclei clusters (Figure 13a,b), (ii) differences between nuclei detection (Figure 13c,d), and (iii) the proposed method eliminates elements that significantly differ from the representative features in the image, such as the average area or circularity used in image filtering (Section 3.1), as illustrated in Figure 13e,f. The similarity between segmentation results and the ground truth was measured for all samples from BCCS and registered in Table 1. As observed, similarity surpasses 0.80 in the metrics when a direct comparison is performed without considering cell cluster separation. When cell clusters are separated, the SDC value is lower since similarity among cell areas decreases because of the division of regions; therefore, sensitivity is also reduced. Nevertheless, accuracy continued showing a high value (0.86). Furthermore, although not considered on the ground truth, cluster separation and elimination of no-representative particles allowed an improved identification of the nuclei, as shown in Figure 13. These are desirable characteristics in histological analysis to determine the degree of cell differentiation in mammary carcinoma.

The pathologist analyzes nuclei variations in size and shape to establish the grade of nuclear pleomorphism in BC. In digital histopathological images, both characteristics are analyzed after nuclei segmentation. Here, two analyses were performed to find differences related to nuclear pleomorphism in (i) cancerous and healthy tissue and (ii) in the three different grades of cell differentiation.

#### 4.2.1. Healthy Tissue vs. Cancerous Tissue

**Figure 14:**
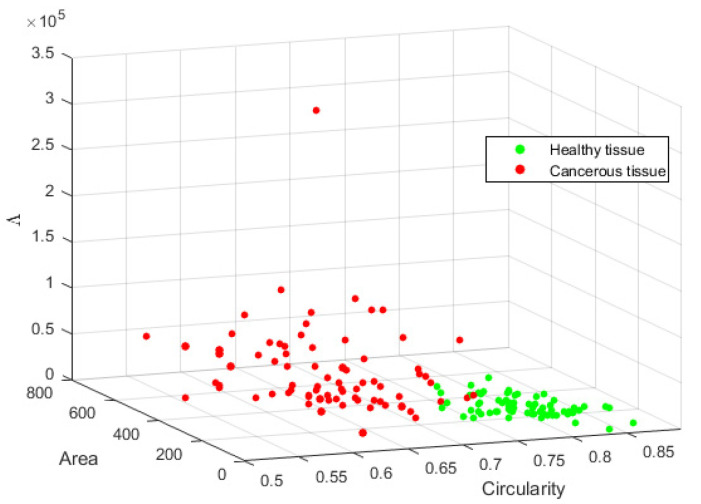
Area-circularity relationship and its dispersion Λ differentiating healthy from cancerous tissues.

For this analysis, segmented nuclei from images of the BreakHis database were obtained for both healthy and cancerous tissue. In healthy tissue, nuclei tend to have high roundness and a more uniform size; the opposite occurs in cancerous tissue. Therefore, it is expected to represent these conditions by quantifying the related features. Then, a multivariate analysis of the area-circularity dispersion of nuclei was performed to know how these two features that allowed segmentation can be used to describe each type of tissue. Given the vectors (A) and (C), containing area and circularity values from nuclei, its covariance matrix M=Cov(A,C) was computed. The covariance matrix allows knowing the dispersion between the features used to describe nuclei. To represent the dispersion of data in *M*, the eigenvalues $\Lambda_{A}$ and $\Lambda_{C}$ were obtained; the general dispersion was represented as $\Lambda =\Lambda_{A}+\Lambda_{C}$. Figure 14 represents the relationship maintained between the analyzed features and their dispersion, where each point represents the mean values from an image. It was observed that nuclei keep high circularity and similar area among the samples in healthy tissue; therefore, dispersion Λ is low. On the other hand, cancerous tissue presents a wider variation in circularity because of nuclei deformation, generating higher Λ between the features. Then, area and circularity measures show suitable features to differentiate the nuclear pleomorphism in healthy and cancerous tissues. Nevertheless, this is a general separation since cancerous tissues are not of a unique type.

#### 4.2.2. Cancerous Differentiation Grades

**Figure 15:**
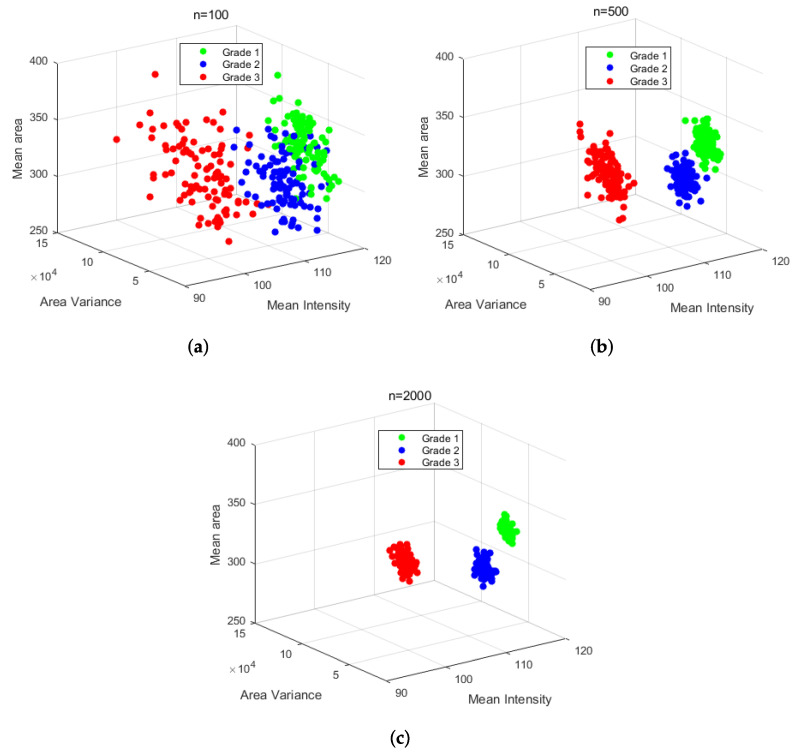
Scatter graphs of the area-circularity relationship variation and mean intensity in 300 samples of the 3 degrees of cell differentiation grade 1, grade 2, and grade 3. (**a**) Graph with random samples of size n = 100, (**b**) graph with random samples of size n = 500, and (**c**) graph with random samples of size n = 2000.

Nuclear pleomorphism in cancerous tissues is divided into three grades of cell differentiation contemplated in the NGS. With the support of two experts pathologists and a doctor, 83 histopathological images of invasive cancer from the BACH database were evaluated and labeled according to the NGS. From this dataset, 13 images are grade 1, 24 images are grade 2, and 46 images are grade 3. The imbalance in the number of images for each grade may generate that their analysis result is not representative enough. Nuclei were extracted individually from all images to generate an equal number of representative subsets and overcome the imbalance. Nuclei were randomly selected and clustered according to the label of the image into subsets of cardinalities n={100,500,2000} for each grade. Thus, each subset was considered a sample of its respective grade. It was assumed that nuclei of the same grade might have similar characteristics then, 100 subsets of each grade were taken, and their values of circularity and area were extracted to be analyzed, as described in Section 4.2.1. It was observed that circularity varies depending on the proportion of healthy and cancerous nuclei in tissue samples of the same grade. On the other hand, it was observed that the mean intensity of nuclei presented lower dispersion than circularity. This behavior may be due to the effect of staining being similar regardless of the state of the cell nucleus. Therefore, the mean intensity and area characteristics were considered to describe each grade, as shown in Figure 15. The sub-sets analysis reflects that the characteristics of each grade show a possible separation when a cardinality of n=100 is considered (Figure 15a). Tests also showed that a higher cardinality (n=500 and n=2000) improves the separation of the NGS grades since statistics are more representative of the sample. Figure 15b,c showed that, although the area varies because of nucleus deformation, mean intensity had low intra-class variation. Thus, the average area and intensity characteristics indicate a significant difference between the three NGS grades of cellular differentiation that can be used for identifying the grade to which a sample belongs.

Based on the relationship between the characteristics and the degree of cell differentiation, it performed a K-Nearest Neighbor (KNN) classification. A total of 200 samples for each degree, previously labeled and clustered with cardinality n=500, were taken to perform automatic classification. Samples were divided in half for the training and test sets. A factor K=5 was used that expresses the number of neighbors considered by KNN. As a result, the classification reached an accuracy of 0.84±0.03 and F1-score of 0.85±0.02 using a 10-fold cross-validation to classify the three degrees of cell differentiation.

### 4.3. Identification of Tubules

The tubules were identified through features obtained from the analysis in Section 3.2.3. Moreover, due to the variations in morphology, tubules classification was addressed using the score measure $C_{s}$ (Equation (6)) from the structural characteristics of the glandular tissue. The $C_{s}$ metric considered the contribution of the different characteristics analyzed. The distance between the centroids of a lumina ellipse and its EC ($l_{cdis}$), as well as the difference in their angles ($l_{\theta }$) are expected to be small, indicating that EC is more likely associated with a lumina (see Figure 10). $C_{s}$ also considers symmetry (*S*) of nuclei distribution around lumina. Although the values of $l_{cdis}$ and $l_{\theta }$ are expected to be small for a lumina, its respective value of *S* is expected to be high. Then, to follow the general trend of values $l_{cdis}$ and $l_{\theta }$ in $C_{s}$, the asymmetry value $l_{A}=\left(1-S\right)*100$ was used instead *S*. Finally, if the minimum threshold of nuclei around lumina ($T_{n}$) is not reached for a candidate, a value $T_{nuclei}=10$ is added as a penalty to the final value. Although $T_{n}$ affects $C_{s}$ its purpose is not to generate a significant change in the final value since lumina could fulfill the other features even being surrounded by a number of nuclei lower than $T_{n}$.

(6) $$C_{s}=T_{nuclei}+l_{cdis}+l_{\theta }+l_{A}$$

**Figure 16:**
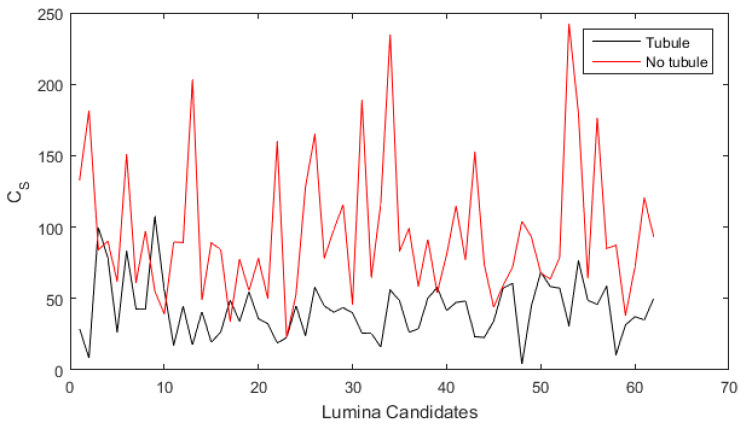
Identification of lumina candidates that correspond to tubules based on the proposed metric $C_{s}$.

The proposed metric was tested on 30 invasive carcinoma images from the BACH database, all containing lumina candidates from which $C_{s}$ scores were obtained. In Figure 16, the plot relates the $C_{s}$ value obtained for 120 candidates extracted from the images and labeled by an expert pathologist as tubule and no tubule. It is observed that lumina candidates labeled as tubules obtained a score $C_{s}=41\pm 19$. In contrast, candidates labeled as no tubule present higher scores and dispersion, i.e., $C_{s}=96\pm 49$. Thus, evidence was found that $C_{s}$ is a metric for differentiating tubules from similar structures by integrating lumina features. By considering the mean and dispersion of $C_{s}$ in lumina candidates corresponding to tubules, it was set that a candidate with values $C_{s}>60$ is unlikely related to a tubule. Under this criterion, it reached an accuracy of 0.83 for tubule identification.

**Figure 17:**
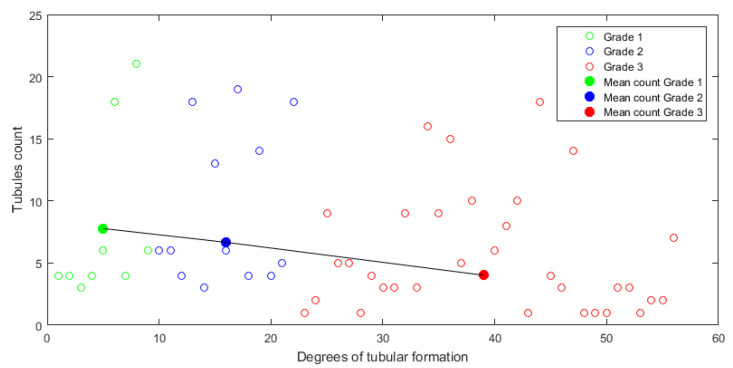
Counting of tubules identified with $C_{S}$ per grade of differentiation.

According to the selection of candidates through $C_{s}$, an analysis of the 56 remaining invasive carcinoma images from the BACH database containing tubules was performed. It is expected that tubule counting is related to the grade of cell differentiation. Since these images were labeled with their differentiation grade (Section 4.2.2), it was possible to emulate the histological evaluation (identification and counting) performed by the pathologist, trying to associate the degree of tubule formation considered in the NGS. Figure 17 shows the tubules counting in obtained for images of each grade (empty circles) and their respective mean counting (filled circles). Due to the lack of a labeled tubule database and the limited number of available images containing tubules per grade, it was not possible to carry out a more extensive analysis for automatic classification or error calculation. However, although there is no significant difference among central tendencies, the proposed metric reflects the tendency of tubule counting to be reduced as the differentiation degree increases in carcinoma images, which matches with the considerations in the NGS.

### 4.4. Discussion

Although several studies have addressed the grade of cell differentiation on histopathological images of breast carcinoma, these do not establish a relationship between their results and the characteristics considered by NGS. Additionally, most methods only considered one of the three characteristics of the tissue. In pathological practice, scores of analyzed structures have equal influence, and their value can affect the final grade determination. Therefore, considering more structures is helpful for the expert. Two main approaches have been reported about the degree of cellular differentiation considering nuclei (Table 2): based on features and automatic classification with DL models. On the one hand, the first approach computes features based on texture, contours, and graphs that, subsequently, were used as an input for a classifier. Petushi et al. [13] achieves an accuracy of 92% differentiating between grades 1 and 3. However, when the comparison considers the three grades, accuracy drops to 72%. Basavanhally et al. [10] also performed a comparison between pairs of grades, obtaining an accuracy of 93% between grades 1 and 3, 72% between grades 1 and 2, and 74% between grades 2 and 3. In addition to texture, graph features were also used in [8] to include information about the way if cell nuclei were arranged in tissue, which is related to cancer progression. The combination of these features allowed differentiation between G1 and G3 with an accuracy of 93%. Similarly, Naik et al. [7] performed a decomposition of low and high-level image information through boundaries segmentation and a template matching with four predefined shapes. Additionally, the spatial relationship between histological structures was considered, reaching an accuracy of 80.5% when comparing grades G1 and G3. As observed, accuracy tends to be higher when extreme grades are compared since their characteristics differ more. Still, the identification task is more complex when adjacent classes or the three classes are considered.

**Table 2:** Comparison of accuracy results for nuclear pleomorphism.

Method	G1 vs. G2	G1 vs. G3	G2 vs. G3	G1 vs. G2 vs. G3	Approach
Petushi et al. [13]	-	92%	-	72%	Texture features
Basavanhally et al. [10]	72%	93%	74%	-	Texture features
Doyle et al. [8]	-	93%	-	-	Texture and graph features
Naik et al. [7]	-	80.5%	-	-	Template matching and morphological features
Cao et al. [12]	74%	90%	76%	-	DL
Wan et al. [11]	77%	92%	76%	69%	DL
Yan et al. [35]	94.1%	97.8%	93.9%	93.4%	DL
Proposed	-	-	-	84%	Morphological and geometrical features

On the other hand, approaches based on deep learning techniques, such as CNNs, identify characteristics from images corresponding to the three grades, i.e., learn to differentiate them. Under this approach, Cao et al. [12] obtained an accuracy of 90% comparing grades 1 and 3, 74% comparing grades 1 and 2, and 76% comparing grades 2 and 3. Wan et al. [11] reported similar accuracy results: 92% for grades 1 and 3, 77% for grades 1 and 2, and 76% for grades 2 and 3; additionally, the evaluation of the three grades obtained 69%. Then, in general, the characterization obtained from both approaches provides lower accuracy results when the three grades are considered. In comparison, the proposed methodology obtained an accuracy of 84% in the three grades of cellular differentiation corresponding to nuclei. Recently, Yan et al. [35] reported the NGNet, a network that allows cell nuclei segmentation and classification. The reported results showed that NGNet reaches 94.1% for grades 1 and 2, 97.8% for grades 1 and 3, 93.9% for grades 2 and 3, and 93.4% among all the grades. This work also mentioned that magnification is an important feature in cell nuclei identification. However, the network was trained only for 20x and 40x, which can be a relevant restriction. In this regard, morphological and geometrical features could provide a more stable description for nuclei classification under different magnification. Moreover, the areas considered important for classification have higher weight when gland-related nuclei (i.e., tubules) are present. In a G3 grade, there is poor or null tubules formation; therefore, this can affect the selection of relevant areas.

In general, both approaches struggle to distinguish among the three degrees of differentiation since it is harder to identify differences. Although with a DL approach higher accuracy is reached, it must be taken into account that to preserve the high performance of the DL model, data must have wide variability including (e.g., staining, amplification, illumination). This is a relevant issue in any medical application and has been analyzed in detail in some works. For instance, in [36] showed that a DL model keeps a good performance only if the data distribution is similar to the training data. Still, it is affected when the tested dataset presents different distribution due to factors, such as a different population, disease characteristics, or imaging systems. In this sense, the feature extraction approach could bring a more stable solution to the description of histopathological structures. The proposed method presents a description based on morphological and geometrical features, which are less variant under staining or amplification conditions and are directly related to structures analyzed by NGS. Moreover, the results were obtained by testing the methodology under the conditions of three different databases, distinguishing among the three different degrees of differentiation, which is the final goal of comparison.

A few recent works have addressed tubular formation. In [13,14], tubules were also identified as high-intensity regions surrounded by nuclei. Although it is mentioned that the number of tubules was considered in the identification of the grades, the analysis of its values was not reported. The main difference with the proposed tubule analysis is the exhaustive evaluation of lumina candidates, considering the general information of the lumina and its spatial and geometric relationship with the surrounding nuclei.

## 5. Conclusions

The proposed methodology focused on identifying and segmenting cell nuclei and tubules from histopathological images, independent of expected changes in conditions, such as staining and magnification. On the one hand, performed nuclei segmentation coincided with the ground truth reaching SDC values of 82%±3. Cluster separation and its relevance in nuclei analysis was also addressed. Furthermore, it was demonstrated that shape characteristics of cell nuclei allow the automatic identification of healthy and cancerous tissue, and among the three grades of differentiation established in the NGS with an accuracy of 84%. On the other hand, based on the analysis performed on the spatial relationship among lumina and nuclei around it, as its geometric description, it was possible to propose a metric to evaluate how likely a lumina candidate corresponds to a tubule. Results from evaluation with $C_{S}$ showed that estimated tubule identification corresponds with the behavior of the differentiation grades described in NGS. Although more extensive research is necessary, the current tubule evaluation reached an accuracy of 83%, providing a starting point. Thus, evidence was found that it is possible to estimate breast carcinoma tissue variations from the analysis of histological images and their digital descriptors, which, in turn, can be associated with the NGS classification.

Moreover, the proposed methodology can then serve as a basis for a digital tool for comparing diagnoses among pathologists or as an educational tool for remote pathology due to the growing need to digitize images for later analysis or discussion of cases to agree on better surgical planning or oncological treatment for patients.

## Figures and Tables

**Table 1:** Evaluation of nuclei segmentation with and without cluster separation.

Metric	No Cluster	Cluster
	Separation	Separation
Coefficient Sørensen–Dice	0.82±0.06	0.79±0.08
Accuracy	0.82±0.08	0.86±0.05
Sensitivity	0.83±0.10	0.76±0.12

mean ± standard deviation.

## Data Availability

Not applicable.
